# The potential predictive value of DEK expression for neoadjuvant chemoradiotherapy response in locally advanced rectal cancer

**DOI:** 10.1186/s12885-018-4048-8

**Published:** 2018-02-06

**Authors:** J. Martinez-Useros, I. Moreno, M. J. Fernandez-Aceñero, M. Rodriguez-Remirez, A. Borrero-Palacios, A. Cebrian, T. Gomez del Pulgar, L. del Puerto-Nevado, W. Li, A. Puime-Otin, N. Perez, M. S. Soengas, J. Garcia-Foncillas

**Affiliations:** 1 Translational Oncology Division, OncoHealth Institute, Health Research Institute - University Hospital “Fundación Jiménez Díaz”-UAM, Av. Reyes Católicos 2, 28040 Madrid, Spain; 2 Department of Pathology, Clinico San Carlos University Hospital, Madrid, Spain; 3 Department of Pathology, University Hospital “Fundación Jiménez Díaz”-UAM, Madrid, Spain; 4 Melanoma Research Group, Spanish National Cancer Research Centre, Madrid, Spain

**Keywords:** DEK, Chemoradiotherapy, Neoadjuvant treatment, Rectal cancer, Predictive biomarker, Complete pathological response

## Abstract

**Background:**

Limited data are available regarding the ability of biomarkers to predict complete pathological response to neoadjuvant chemoradiotherapy in locally advanced rectal cancer. Complete response translates to better patient survival. DEK is a transcription factor involved not only in development and progression of different types of cancer, but is also associated with treatment response. This study aims to analyze the role of DEK in complete pathological response following chemoradiotherapy for locally advanced rectal cancer.

**Methods:**

Pre-treated tumour samples from 74 locally advanced rectal-cancer patients who received chemoradiation therapy prior to total mesorectal excision were recruited for construction of a tissue microarray. DEK immunoreactivity from all samples was quantified by immunohistochemistry. Then, association between positive stained tumour cells and pathologic response to neoadjuvant treatment was measured to determine optimal predictive power.

**Results:**

DEK expression was limited to tumour cells located in the rectum. Interestingly, high percentage of tumour cells with DEK positiveness was statistically associated with complete pathological response to neoadjuvant treatment based on radiotherapy and fluoropyrimidine-based chemotherapy and a marked trend toward significance between DEK positiveness and absence of treatment toxicity. Further analysis revealed an association between DEK and the pro-apoptotic factor P38 in the pre-treated rectal cancer biopsies.

**Conclusions:**

These data suggest DEK as a potential biomarker of complete pathological response to treatment in locally advanced rectal cancer.

## Background

Colorectal cancer is one of the most common gastrointestinal malignant tumours in the world and has one of the highest rates of morbidity and mortality worldwide. It is not only the third most common malignancy in United States but also the third leading cause of cancer-related deaths [1]. Rectal cancer accounts for between 27% and 58% of all cases of colorectal cancer, with variations attributable to the cancer registry studied and the method used to classify rectosigmoid tumours [2]. Of the 304,930 new cases of digestive-tract cancer diagnosed in 2016 in the United States, 39,220 were rectal, with higher incidence seen among males than females (23,110 vs. 16,110) [1]. Further information about the global incidence of rectal cancer can be obtained from the World Health Organization (WHO)-GLOBOCAN [3, 4].

A distinction must be made between rectal and colon carcinoma, as rectal cancer has a distinct dissemination pattern. Furthermore, surgical resection is the mainstay of curative treatment for rectal adenocarcinomas [5]. Colon carcinoma is located in the peritoneal cavity, an area that is highly accessible and facilitates surgical intervention with wide resection margins. In contrast, rectal cancer is located extraperitoneally, within the pelvis, thus it makes harder the surgical resection that in most of cases involve low anterior or abdominoperineal resection. Some rectal tumours are superficial (T0/T1) and small enough (< 3 cm) to be successfully resected by local excision. However, most patients have more deeply invasive tumours that are adherent or fixed to adjoining structures (e.g., sacrum, pelvic sidewalls, prostate, or bladder) that requires more extensive resection [6].

Rectal tumours tend toward local recurrence, and surgery alone only provides a high cure rate for patients with early-stage disease [7]. In fact, the five-year survival rate for patients with stage I tumours is around 80 to 90%, while this rate is below 80 for those with stage II or III disease [8].

To increase long-term survival, the Swedish Study Group has introduced neoadjuvant treatment for locally advanced tumours based on chemotherapy combined with radiation [9]. The effects of chemoradiotherapy are the results of DNA damage produced directly by ionizing radiations; or indirectly, by the action of chemical radicals generated from ionization [10]. Chemoradiotherapy improves survival rates and local recurrence by reducing tumour size and stage, and also has the ability to achieve pathologic downstaging [11, 12]. For these reasons, neoadjuvant chemotherapy is the standard of care for stage II–III rectal tumours, not only to increase the effectiveness of radiotherapy but also to attain negative surgical margins [13] and enhance the possibility for sphincter-preserving surgery [14]. As described by Ryan et al., tumour regression grade is a useful method of scoring pathologic response to chemoradiotherapy in rectal carcinomas [15]. However, complete pathological response has been reported in only 10% to 30% of patients, and around 40% show partial or no response [16].

To predict response to neoadjuvant treatment, translational research has focused on the search for potential biomarkers of response to preoperative treatment [17–19].

DEK was identified fusioned with the CAN nucleoporin due to the translocation t (6;9) in a subtype of acute myeloid leukemia [20]. DEK is overexpressed in multiple neoplasms, including bladder cancer [21], breast cancer [22], glioblastoma [23], hepatocellular carcinoma [24], melanoma [25], retinoblastoma [26, 27], and other types, such as oral, ovarian, or uterine-cervical cancer [28–31].

Functionally, DEK is involved in the DNA damage repair machinery from the interaction with PARP-1 [32], suppresses apoptosis, senescence, differentiation, and promotes cell transformation both in vitro and in vivo [33–35]. Our group has previously associated DEK expression with adjuvant-treatment response in colorectal cancer [36]. Here, we observed a significant increase in apoptotic cells after the combination of irinotecan treatment and DEK knock-down, compared to those treated with irinotecan or DEK knock-down individually. However, this effect was not observed with 5FU or oxaliplatin treatments alone or in combination with DEK knock-down [36].

DEK has also been described to have a high statistical power to predict pathological complete response for neoadjuvant chemotherapy in breast cancer [37].

Therefore, our hypothesis to link DEK with neoadjuvant therapy in rectal cancer has been based on the above-mentioned reports that associated DEK with treatment response.

This study aimed to explore the precise role of DEK as a novel biomarker of pathologic response in rectal adenocarcinoma. To achieve this, 74 biopsies obtained from pre-treated locally advanced rectal-adenocarcinoma patients were immunostained with DEK. Association with neoadjuvant chemoradiotherapy response was assessed in light of these findings.

## Methods

### Patient samples

The follow-up of 91 consecutive patients with stage II or stage III rectal adenocarcinoma according to American Joint Committee on Cancer [38] who underwent standardized neoadjuvant chemoradiotherapy followed by total mesorectal excision, from December 2006 to January 2014, were reviewed for the study. However, only those patients with available endoscopic biopsies for immunohistochemical analysis were selected for this study. A total of 74 patients with locally advanced rectal adenocarcinoma, from General and Digestive-Tract Surgery Department of University Hospital Fundación Jiménez Díaz were assessed for eligibility.

Sixty-three percent of the rectal tumours included in the study were determined to be of a high grade based on the recommendations of the College of American Pathologists [39]. Magnetic resonance imaging (MRI), computed tomography, endorectal ultrasound, and/or endoscopy revealed a high prevalence of stage III tumours (93%). The criteria published by Ryan et al. were applied to classify patients according to response to neoadjuvant treatment [15]. According to this classification system, complete pathological response was indicated by an absence of tumour cells; partial pathologic response by fibrosis with presence of isolated tumour cells; and minimum pathologic response by tumour nests outgrown by fibrosis or no tumour kill. T- and N-downstaging were also assessed. Radiotherapy administered as neoadjuvant treatment was dosed over 28 sessions (45 Gy to the pelvic area and 50.4 Gy to the tumour area).

### Tissue microarray

Samples from 74 patients were used to construct a paraffin block containing 148 cores (2 cores per patient) to allow for immunohistochemistry analysis. A hollow needle (MTA-1 tissue arrayer, Beecher Instruments, Sun Prairie, USA) was used to perform a punch biopsy from pre-selected tumour areas in paraffin-embedded (FFPE) tissues. These tissue cores were then inserted in a recipient paraffin block. Sections from this FFPE block were cut using a microtome and mounted on a microscope slide to be analyzed by immunohistochemistry.

### Immunohistochemistry and quantification

Staining was conducted in 2-μm sections. Slides were deparaffinized by incubation at 60 °C for 10 min and then incubated with PT-Link (Dako, Denmark) for 20 min at 95 °C in a high pH-buffered solution. To block endogenous peroxidase, holders were incubated with peroxidase blocking reagent (Dako, Denmark). Biopsies were stained for 20 min with a 1:50 dilution of DEK antibody (610,948, BD Biosciences) and with 1:150 of phospho-P38 (ab38238, Abcam) followed by incubation with anti-Ig horseradish peroxidase-conjugated polymer (EnVision, Dako, Denmark) to detect antigen-antibody reaction. A single human normal rectum tissue was used as a positive control for immunohistochemical staining. Sections were then visualized with 3,3′-diaminobenzidine as the chromogen for 5 min and counterstained with hematoxylin. Photographs were taken with a stereo microscope (Leica DMi1, Wetzlar, Germany). Immunoreactivity was quantified by two independent pathologists as the percentage of positive stained cells over the total number of tumour cells. Positiveness was defined as medium to high DEK expression levels according to *The Human Protein Atlas* (http://www.proteinatlas.org) and quantification of each biopsy was calculated using the average of both cores.

### Statistical analysis

The association between DEK expression (categorized as low or high percentage of positive stained cells) and clinicopathologic variables, including pathologic response, was evaluated by *Fisher’s exact* or Chi-square (χ^2^) test. χ^2^ test was used to analyze the relationship between DEK expression and clinicopathologic parameters. *Fisher’s exact* test was used when one or more variable had a frequency of five or less. Association between phospho-P38 expression (categorized as low or high percentage of positive stained cells) with pathologic response was assessed by *Fisher’s exact* test. Association between DEK and phospho-P38 expression was analysed by χ^2^ test. *P* values ≤0.05 were considered significant. Analysis was performed with the IBM SPSS program, version 20.0.

## Results

### Patient characteristics

The clinical features of the resected rectal-cancer patients are summarized in Table 1. The median age of the patients was 72 years (range 46–89 years), and male population has higher incidence (*n* = 45; 61%) with good performance status (ECOG 0) (*n* = 41; 55%).

**Table 1:** Clinico-pathologic characteristics of rectal cancer patients

Characteristics	Patients (*N* = 74)
Median age-years (range)	72 (46–89)
> 60 years	60 (81%)
< 60 years	14 (19%)
Sex	
Male	45 (61%)
Female	29 (39%)
ECOG	
0	41 (55%)
1	31 (42%)
2	2 (3%)
Status	
Death	7 (10%)
Alive without disease	59 (78%)
Alive with disease	7 (10%)
N/A	1 (1%)
T Downstaging	
0	28 (38%)
1	39 (53%)
N/A	7 (9%)
N Downstaging	
0	20 (27%)
1	47 (64%)
N/A	7 (9%)
Grade	
Low	19 (26%)
High	47 (63%)
N/A	8 (11%)
Stage	
II	4 (6%)
III	69 (93%)
N/A	1 (1%)
Neoadjuvant Treatment	
RT + Fluoropyrimidines based	73 (99%)
Other	1 (1%)
Treatment toxicity	
Yes	30 (41%)
No	44 (59%)
Pathological Response	
Complete	9 (12%)
Partial	27 (37%)
Minimun	38 (51%)
DEK	
Low	26 (35%)
High	48 (65%)

*N/A* not available, *RT* Radiotherapy

Neoadjuvant treatment was based on fluoropyrimidines (5FU or FOLFOX) and combined with radiotherapy was administered in 73 patients (99%). The majority of patients did not present treatment toxicity (*n* = 44; 59%). Concerning pathological response, complete response was achieved in 9 patients (12%) and partial and minimum response in 27 patients (37%), and 38 patients (51%) respectively.

### High DEK expression associated with complete response to neoadjuvant chemoradiotherapy

**Fig. 1:**
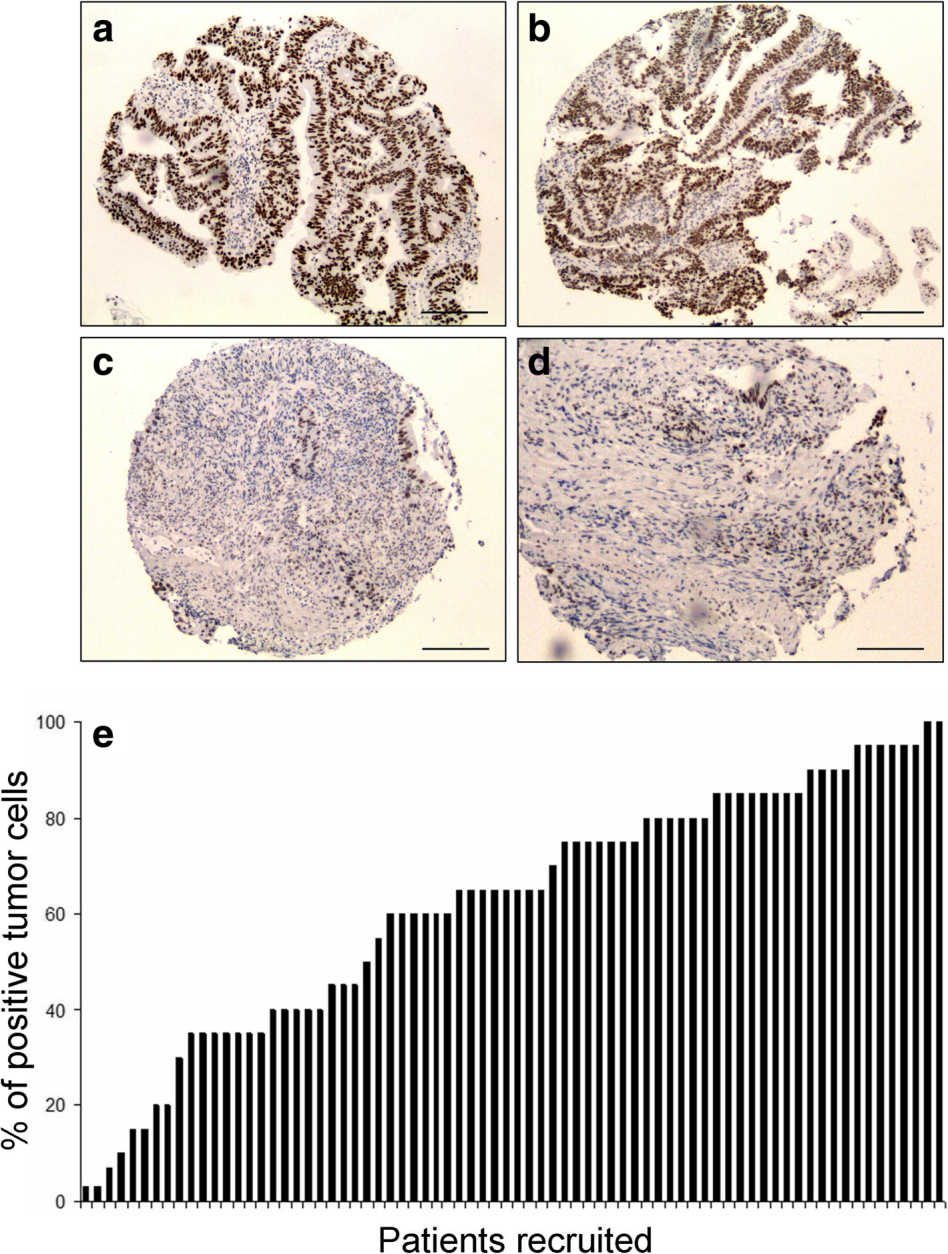
Differential pattern of DEK positive stained cells of locally advanced rectal tumours. **a** and **b** representative images of tumour samples with high percentage of DEK positive stained cells. **c** and **d** representative images of tumour samples with low percentage of DEK positive stained cells. Scale bar is 50 μm. **e** Histogram of patient samples according to percentage of DEK positive tumour cells

Based on our previous reports [36], we hypothesized that DEK could be related to neoadjuvant response and serve as a predictive biomarker in patients with rectal adenocarcinoma prior to surgery. For this purpose, a tissue microarray was constructed and stained to quantify the percentage of DEK positive cells over the total number of tumour cells. All samples were obtained before the patients received neoadjuvant treatment. After immunohistochemical staining, the biopsies were observed to have nuclear localization and DEK stained only tumour cells (Fig. 1a to d). Distribution of samples according to the percentage of positive tumour cells staining showed a uniform cumulative distribution (Fig. 1e). The biopsies were then stratified into low or high DEK expression using the mean percentage of positive stained tumor cells as a cut-off point. The results showed that 9 (19%) patients out of the 45 patients with high DEK expression achieved a complete response to neoadjuvant treatment; while none of those with low DEK expression obtained a complete response. In fact, all patients who showed complete response (*n* = 9) had high DEK expression. Moreover, 82% of patients (*n* = 39) with high expression achieved partial or minimal response, while all patients (*n* = 26; 100%) with low DEK expression achieved partial or none response (Table 2). Statistical analysis showed significant differences between both groups of response to neoadjuvant chemoradiotherapy (complete vs. partial or minimal) and the low or high DEK expression (*Chi-squared: P =* 0,018*; Fisher’s exact: P =* 0,023) (Table 2).

**Table 2:** Statistical association between neoadjuvant treatment response and low- or high-percentage of DEK positive tumor cells

	Treatment Response			
DEK	No. Complete (% of DEK subpopulation)	No. Partial or minimum (% of DEK subpopulation)	*P* (chi-square)	*P* (Fisher)
High	9	39		
* n* = 48	(19%)	(82%)		
			0,018	0,023
Low	0	26		
* n* = 26	(0%)	(100%)		

*No* Number of patients

Further analysis revealed no statistical association between DEK expression and the rest of the clinicopathologic variables studied, including gender (*P* = 0.553), age (*P* = 0.758), T-downstaging (*P* = 0.840), N-downstaging (*P* = 0.626), grade (*P* = 0.312), ECOG (*P* = 0.843), status (*P* = 0.544), tumour size (*P* = 0.703), and stage (*P* = 0.613). Concerning treatment toxicity, a considerable trend was observed between high DEK expression and the absence of treatment toxicity (*P =* 0.086*)* (Table 3).

**Table 3:** Statistical association between low- or high-percentage of DEK positive stained tumor cells and clinico-pathological parameters

	DEK		
Parameter	Low	High	*P*
Gender			0.553
Male	17	28	
Female	9	20	
Age			0.758
< 60 years	4	10	
> 60 years	22	38	
T_Downstaging			0.840
No	10	18	
Yes	13	26	
N_Downstaging			0.626
No	6	14	
Yes	17	30	
Grade			0.312
Low	9	10	
High	16	31	
Treatment toxicity			0.086
Yes	14	16	
No	12	32	
ECOG			0.843
0	14	27	
1–2	12	21	
Status			0.544
Alive with disease or death	6	8	
Alive without disease	20	40	
Tumor size			0.703
< 3 cm	2	6	
> 3 cm	24	41	
Stage			0.613
II	2	2	
III	24	45	

### DEK expression associated with phospho-P38 expression in pre-treated rectal cancer biopsies

P38 is an important component of the mitogen-activated protein kinases (MAPK) [40] and plays a central role in cell proliferation and apoptosis in multiple neoplasias [41]. Furthermore, P38 has been recently associated to chemotherapy response in colorectal cancer [42]. Therefore, we quantified the immunoreactivity of the active form of P38 (phospho-P38) in all rectal cancers biopsies by immunohistochemistry. Phospho-P38 expression was then categorized as low or high according to median percentage of positive stained tumor cells as cut-off point. Although we did not find statistically significant association between phospho-P38 expression and pathological response to neoadjuvant treatment (*P* = 0.296; data not shown), a direct association was found between phospho-P38 and DEK expression (*P* = 0.027; Table 4). In fact, seven patients of whom showed not only complete response but also high DEK expression (*n* = 9) revealed high expression of phospho-P38, while two patients presented low phospho-P38 expression.

**Table 4:** Statistical association between phospho-P38 and DEK positiveness in rectal cancer patients treated with neoadjuvant chemoradiotherapy

DEK \ phospho-P38	Low	High	Total	*P* (chi-square)
Low	15	15	30	
(%)	(20%)	(20%)	(40%)	
High	11	33	44	
(%)	(15%)	(45%)	(60%)	
Total	26	48	74	0,027
(%)	(35%)	(65%)	(100%)

**Table 5:** Dataset of patient biopsies recruited in the study

Biopsy	Age	ECOG_PS	Status	T-Downstaging	N-Downstaging	Grade	Stage	Neoadjuvant treatment	Treatment toxicity	Pathological Response	Tumor size	DEK (% positive tumor cells)	Phospho-P38 (% positive tumor cells)
1	> 70	1	alive without disease	0	1	High	III	RDT + FOLFOX	No	Partial	> 3 cm	3	35
2	> 60	1	alive without disease	0	1	High	III	RDT + 5FU	No	Minimum	> 3 cm	3	35
3	> 70	0	Death	1	0	High	III	RDT + FOLFOX	Yes	Minimum	< 3 cm	7	65
4	> 70	0	alive without disease	0	1	Low	III	RDT + 5FU	No	Minimum	> 3 cm	10	80
5	> 60	0	alive with desease	0	1	Low	III	RDT + FOLFOX	Yes	Minimum	> 3 cm	15	45
6	> 70	0	alive without disease	1	1	High	III	RDT + FOLFOX	Yes	Partial	> 3 cm	15	80
7	> 40	0	alive with desease	0	0	High	III	RDT + 5FU	Yes	Partial	> 3 cm	20	25
8	> 40	1	alive with desease	1	0	High	II	RDT + 5FU	Yes	Minimum	> 3 cm	20	25
9	> 70	0	alive without disease	1	1	Low	III	RDT + 5FU	No	Partial	> 3 cm	30	45
10	> 70	0	alive without disease	1	1	High	III	RDT + FOLFOX	Yes	Minimum	> 3 cm	35	55
11	> 70	0	alive without disease	N/A	N/A	High	III	RDT + FOLFOX	No	Minimum	> 3 cm	35	10
12	> 50	0	alive without disease	0	1	High	III	RDT + 5FU	Yes	Minimum	> 3 cm	35	70
13	> 70	0	alive without disease	1	1	High	III	RDT + 5FU	No	Partial	> 3 cm	35	25
14	> 50	0	alive without disease	1	1	Low	III	RDT + 5FU	No	Minimum	> 3 cm	35	25
15	> 70	1	alive without disease	1	1	High	III	RDT + 5FU	Yes	Minimum	< 3 cm	35	90
16	> 60	1	alive without disease	1	1	Low	III	RDT + 5FU	No	Partial	> 3 cm	35	90
17	> 60	0	N/A	0	0	High	III	RDT + FOLFOX	Yes	Minimum	> 3 cm	40	5
18	> 80	0	Death	1	0	High	III	RDT + 5FU	Yes	Partial	> 3 cm	40	90
19	> 70	1	alive without disease	0	1	Low	III	RDT + 5FU	Yes	Minimum	> 3 cm	40	85
20	> 60	0	alive without disease	0	1	High	III	RDT + 5FU	No	Minimum	> 3 cm	40	65
21	> 70	2	alive without disease	1	0	N/A	II	RDT + 5FU	Yes	Minimum	> 3 cm	40	100
22	> 70	1	Death	0	1	High	III	RDT + 5FU	Yes	Partial	> 3 cm	45	65
23	> 80	1	alive without disease	N/A	N/A	High	III	RDT + 5FU	No	Partial	> 3 cm	45	40
24	> 70	1	alive without disease	N/A	N/A	Low	III	RDT + 5FU	Yes	Partial	> 3 cm	45	80
25	> 80	1	alive without disease	1	1	Low	III	RDT + 5FU	No	Partial	> 3 cm	50	80
26	> 70	1	alive without disease	1	1	Low	III	RDT + 5FU	No	Partial	> 3 cm	55	80
27	> 50	0	alive without disease	1	1	High	III	RDT + FOLFOX	Yes	Minimum	> 3 cm	60	80
28	> 80	1	alive without disease	N/A	N/A	High	N/A	RDT + 5FU	No	Complete	> 3 cm	60	60
29	> 80	1	alive without disease	1	1	High	III	RDT + FOLFOX	Yes	Minimum	> 3 cm	60	75
30	> 60	0	alive without disease	0	0	High	III	RDT + 5FU	No	Minimum	> 3 cm	60	40
31	> 50	1	alive with desease	1	1	High	III	RDT + 5FU	No	Complete	< 3 cm	60	100
32	> 60	0	alive without disease	N/A	N/A	High	III	RDT + 5FU	No	Complete	> 3 cm	60	45
33	> 40	0	alive without disease	0	0	High	III	RDT + FOLFOX	Yes	Minimum	> 3 cm	65	100
34	> 80	0	alive without disease	1	1	High	III	RDT + 5FU	Yes	Partial	> 3 cm	65	75
35	> 80	0	alive without disease	1	0	High	III	RDT + 5FU	No	Partial	> 3 cm	65	75
36	> 80	1	alive without disease	1	1	N/A	III	RDT + 5FU	Yes	Partial	< 3 cm	65	90
37	> 70	0	alive without disease	1	1	N/A	III	RDT + 5FU	No	Complete	> 3 cm	65	90
38	> 70	1	alive without disease	1	1	Low	III	RDT + 5FU	No	Minimum	> 3 cm	65	15
39	> 60	0	alive without disease	1	1	High	III	RDT + 5FU	No	Partial	> 3 cm	65	80
40	> 60	1	alive without disease	1	0	High	III	RDT + 5FU	No	Partial	> 3 cm	65	50
41	> 40	0	alive without disease	1	1	Low	III	RDT + 5FU	Yes	Partial	> 3 cm	70	60
42	> 80	1	alive without disease	1	1	High	III	RDT + 5FU	No	Complete	> 3 cm	75	80
43	> 70	0	alive with desease	1	0	Low	III	RDT + FOLFOX	No	Minimum	< 3 cm	75	90
44	> 70	1	alive without disease	N/A	N/A	High	III	RDT + 5FU	No	Minimum	> 3 cm	75	75
45	> 40	0	alive without disease	1	1	N/A	III	RDT + 5FU	No	Complete	> 3 cm	75	95
46	> 60	0	alive without disease	1	1	Low	III	RDT + 5FU	Yes	Complete	< 3 cm	75	90
47	> 80	1	alive without disease	0	1	High	III	RDT + 5FU	Yes	Minimum	> 3 cm	75	95
48	> 60	1	alive with desease	0	1	High	III	RDT + 5FU	Yes	Complete	> 3 cm	75	70
49	> 70	0	Death	1	1	Low	III	others	Yes	Minimum	< 3 cm	80	3
50	> 80	1	Death	0	0	High	III	RDT + FOLFOX	Yes	Minimum	> 3 cm	80	35
51	> 70	0	alive without disease	0	0	High	III	RDT + 5FU	No	Partial	> 3 cm	80	100
52	> 60	0	alive without disease	0	1	High	III	RDT + 5FU	Yes	Partial	< 3 cm	80	95
53	> 70	1	alive without disease	1	0	N/A	III	RDT + 5FU	No	Partial	> 3 cm	80	30
54	> 60	0	alive without disease	0	0	N/A	II	RDT + 5FU	No	Partial	> 3 cm	80	10
55	> 70	1	alive without disease	1	1	N/A	III	RDT + FOLFOX	No	Partial	> 3 cm	85	90
56	> 80	1	alive without disease	0	1	High	III	RDT + 5FU	Yes	Minimum	> 3 cm	85	45
57	> 50	0	alive without disease	1	1	Low	III	RDT + 5FU	No	Partial	> 3 cm	85	75
58	> 50	1	alive without disease	0	0	High	III	RDT + 5FU	No	Minimum	> 3 cm	85	85
59	> 60	0	alive without disease	1	1	Low	III	RDT + 5FU	No	Partial	> 3 cm	85	30
60	> 80	1	alive without disease	1	1	High	III	RDT + 5FU	No	Minimum	> 3 cm	85	80
61	> 60	0	alive without disease	1	1	Low	III	RDT + 5FU	No	Partial	> 3 cm	85	0
62	> 50	0	alive without disease	N/A	N/A	Low	III	RDT + FOLFOX	No	Minimum	> 3 cm	85	100
63	> 70	1	alive without disease	1	1	High	III	RDT + 5FU	No	Minimum	N/A	90	95
64	> 70	0	alive without disease	0	1	High	III	RDT + 5FU	No	Minimum	> 3 cm	90	85
65	> 70	1	Death	0	0	High	III	RDT + 5FU	Yes	Minimum	> 3 cm	90	45
66	> 50	0	alive without disease	1	1	Low	III	RDT + 5FU	No	Minimum	> 3 cm	90	55
67	> 60	2	Death	0	0	High	III	RDT + 5FU	Yes	Minimum	> 3 cm	95	100
68	> 70	0	alive without disease	1	0	N/A	II	RDT + 5FU	No	Minimum	> 3 cm	95	85
69	> 60	0	alive without disease	0	1	High	III	RDT + 5FU	No	Partial	> 3 cm	95	80
70	> 60	0	alive without disease	0	1	High	III	RDT + 5FU	Yes	Minimum	> 3 cm	95	90
71	> 70	0	alive with desease	0	1	High	III	RDT + 5FU	No	Minimum	> 3 cm	95	75
72	> 70	0	alive without disease	1	0	High	III	RDT + 5FU	No	Complete	> 3 cm	95	75
73	> 80	1	alive without disease	0	1	High	III	RDT + 5FU	No	Minimum	> 3 cm	100	75
74	> 50	1	alive without disease	0	1	High	III	RDT + 5FU	No	Minimum	> 3 cm	100	80

*N/A* Not available, *RDT* radiotherapy

These results suggest that high DEK expression in tumour biopsies could be used as a potential biomarker of pathological response that follows neoadjuvant therapy in rectal cancer. Moreover, the association between DEK and phospho-P38 expression supports and provides a highly robust predictive model of cell-death revealed by the complete response to neoadjuvant treatment.

## Discussion

Neoadjuvant chemoradiotherapy is the standard care approach for stage II and III rectal-cancer patients. The aim of this treatment is to achieve pathologic downstaging and complete response. Therefore, extensive investigation is currently being devoted to biomarkers that predict response to neoadjuvant treatment. Genetic profiling platforms have become a useful tool for analyzing DNA, RNA, and other factors that may or may not be translated into protein, such as miRNA. In the era of genomics, transcriptomics, and proteomics, these methodologies have helped elucidate potential biomarkers of treatment response in rectal cancer [17, 43–47]. DNA microarrays have been used to differentiate rectal-cancer patients into responders and non-responders. A study using DNA microarrays to assess 17 rectal-cancer samples discovered 17 genes differentially expressed between responders and non-responders [44]. Some of these genes included *MMP*, *NFKB2*, *TGFB1*, *TOP1*, and *ITGB1* [44]. The most highly overexpressed gene, *MMP7*, was validated by immunohistochemistry, and it was found that none of the non-responders (*n* = 7) overexpressed the gene. However, only four of the responders (*n* = 10) overexpressed MMP7 [44]. Palma et al. analyzed the gene-expression profiles of 26 pre-treatment biopsies by expression microarray and demonstrated that high levels of Gng4, c-Myc, Pola1, and Rrm1 expression were significant factors when predicting neoadjuvant response in rectal cancer [45]. Others studies with 23 patient samples [17] and with 43 patient samples [43] revealed 54 and 43 differentially expressed genes, respectively, though no concordance was found between both studies. Some studies based on miRNA microarrays revealed higher miR-223 levels in responders compared to non-responders, one in a cohort of 43 rectal-cancer patients [46], and a more recent in a cohort of 59 patients [47].

Post-translational modifications may affect the concordance between gene-expression profile and protein-expression pattern, which could lead to controversial results. Proteins are the main agents in biologic pathways, and thus the results of protein-expression analysis may be the key to treatment decision-making. Regarding the prediction of response to chemoradiotherapy in rectal cancer by immunohistochemistry, Kuremsky et al. reported that the most commonly biomarkers evaluated were p53, EGFR, TYMS, Ki-67, p21, BCL-2, and BAX [48].

High DEK expression has been described previously by our group as a crucial event for aggressive tumour phenotype and as a biomarker for poor response to irinotecan in metastatic colorectal cancer [36]. In the present study, high DEK expression was related to pathological response in 74 locally advanced rectal adenocarcinomas. This enabled us to establish a new model based on DEK expression that was statistically associated with complete pathological response. Here, it is supported that rectal cancer patients with high DEK expression have a 19% probability to achieve complete response. Otherwise, low DEK expression predicts lack of complete response to neoadjuvant treatment. Moreover, the fact that DEK expression associated with the pro-apoptotic factor P38 supports the role of DEK as a predictive biomarker for pathological complete response to chemoradiotherapy prior to surgery in rectal cancer patients.

The findings showed in the present study seem to disagree with those obtained in our previous work with colorectal cancer [36]. However, our previous research was performed with stage IV colorectal cancer samples, while the present work only focused on stage II–III rectal tumours that only represent a part of colorectal tumors. Moreover, the potential effect of DEK in our previous study to predict irinotecan response was not observed with 5FU or oxaliplatin, drugs used in the present study to evaluate pathological response. Indeed, DEK has also been related to neoadjuvant treatment response in breast cancer, independently of estrogen-receptor status [49]. Consequently, our study agree with Witkiewicz et al., who reported a strong association between high DEK expression and a low residual cancer burden, indicative of preferred response to neoadjuvant chemotherapy [49].

## Conclusions

This retrospective study supports DEK as a potential predictive biomarker for neoadjuvant treatment response in rectal cancer. Moreover, the methodology performed here is easy and reproducible enough to be implemented in the routine clinical practise.

Although further research is needed, this preliminary study could be used to prospectively validate the predictive value of DEK expression in rectal and other types of tumours prior neoadjuvant treatment.

## Abbreviations

5FU: 5-Fluorouracil
BAX: BCL2-associated X protein
BCL-2: B-cell lymphoma 2
c-MYC: c-myelocytomatosis viral oncogene
DEK: DEK proto-oncogen
ECOG: Eastern cooperative oncology group
EGFR: Epidermal growth factor receptor
FFPE: Formalin-fixed paraffin-embedded
FOLFOX: Folinic acid + 5-Fluorouracil + Oxaliplatin
GNG4: G protein subunit gamma 4
Gy: Gray
ITGB1: Integrin subunit beta 1
Ki-67: Marker of proliferation Ki-67
MAPK: Mitogen-activated protein kinases
MMP: Matrix metallopeptidases
MRI: Magnetic resonance imaging
N/A: Not available
NFKB2: Nuclear factor of kappa light polypeptide gene enhancer in B-cells 2
POLA1: Polymerase (DNA) alpha 1
RRM1: Ribonucleotide reductase M1
RT: Radiotherapy
TGFB1: Transforming growth factor beta 1
TOP1: Topoisomerase (DNA) I
TYMS: Thymidylate synthetase
